# Influence of Cooking and Ingredients on the Antioxidant Activity, Phenolic Content and Volatile Profile of Different Variants of the Mediterranean Typical Tomato *Sofrito*

**DOI:** 10.3390/antiox8110551

**Published:** 2019-11-14

**Authors:** Ana Beltrán Sanahuja, Saray López De Pablo Gallego, Salvador E. Maestre Pérez, Arantzazu Valdés García, María Soledad Prats Moya

**Affiliations:** Analytical Chemistry, Nutrition and Food Science Department, University of Alicante, P.O. Box 99, E-03080 Alicante, Spain; ana.beltran@ua.es (A.B.S.); saraylopezpg@gmail.com (S.L.D.P.G.); salvador.maestre@ua.es (S.E.M.P.); arancha.valdes@ua.es (A.V.G.)

**Keywords:** sofrito, tomato sauce, experimental design, volatile composition, antioxidant activity, total phenolic content

## Abstract

In this study, six different sofrito formulations were compared with the raw recipe for total phenolic content (TPC), antioxidant activity tested by 2,2-diphenyl-1-picrylhydrazyl (DPPH), ferric-reducing antioxidant power (FRAP) and 2,2-azinobis (3-ethylbenzothiazoline-6-sulfonic acid) diammonium salt (ABTS) methods. The volatile profile was also obtained by the headspace solid-phase microextraction-gas chromatography mass spectrometry (HS-SPME-GC–MS) procedure. The cooking process and the addition of herbs, and garlic improved the final content of antioxidant compounds compared to the basic recipe and the raw ingredients. The total volatile content was higher in the samples that contained rosemary and thymus. Some of the volatiles had proven antioxidant properties and for that reason the *sofrito* with rosemary with the higher volatile content was also the one with the higher antioxidant capacity and TPC. In conclusion, as well as the processing technique, the addition of selected typical Mediterranean herbs apart from given flavour can contribute to improving the nutritional antioxidant profile of dishes and be used as a natural method to increase the shelf-life of preparation.

## 1. Introduction

Tomato *sofrito* consists of a sauce made with tomatoes, onions, and garlic simmered with olive oil. Additionally, other components can also be added as vegetables, spices and herbs. Recently, *sofrito* was included in a reduced list of recommended foods that should be consumed at least 2 times a week for a healthiest Mediterranean diet due to its high content of available bioactive compounds [1].

Cooking techniques together with the proportion of ingredients may modify the bioavailability of bioactive compounds [2]. *Sofrito* sauce (S) has a natural gel texture as during the cooking process the pectins and hemicelluloses from vegetables are softened and consequently the vegetable cell walls are disrupted. Therefore, free pectins escape into the fluid phase thickening the sauce. At the same time, the inner contents of the cells become more accessible such as, for example, polyphenols and other flavouring molecules such as organic acids, sugars and volatile compounds [3]. This effect is negative from a nutritional point of view on those thermosensitive antioxidants like vitamin C but at the same time, the extractability of bounded polyphenols and flavonoids is improved as they become more available [4,5,6]. In this sense, a recent study concluded that the use of onions among the ingredients of the *sofrito* combined with an adequate simmering process improves the bioavailability of lycopene in tomato products [2]. However, *sofrito* contains other valuable compounds which can contribute to healthy properties of the preparation such as polyphenols, ascorbic acid, and vitamin E.

The determination of antioxidant capacity and total polyphenol content assays are frequently found in literature, their sensitivity and selectivity being different, although the principle of working is similar. Studies based on antioxidant capacity in fresh and industrial processed tomato using different types of assays such as the 2,2-diphenyl-1-picrylhydrazyl (DPPH) radical scavenging activity [6,7], total antioxidant activity (TEAC) [4], ferric-reducing antioxidant power (FRAP) and diammonium salt (ABTS) radical cation scavenging assay [7] can be found in the literature. These studies are focused on industrially processed tomatoes [5] and derivatives but information on the influence on the change of one ingredient on the antioxidant and volatile contents in homemade *sofrito* is actually scarce.

*Sofritos* are usually employed in the Mediterranean countries to introduce characteristic flavours to the food elaborations, indirectly contributing to important bioactive components to the typical dishes. Thereby in Spain and other Mediterranean countries, *sofritos* are an essential step in the elaboration of typical dishes such as paella, legumes, pasta, etc. Regarding its volatile profile, thousands of compounds have been described in tomato sauces using the headspace gas chromatography technique (HS-GC) [8]. Nevertheless, besides the flavour typically found in fresh vegetables, new volatiles are generated during the cooking process mainly as products of oxidation of carotenoids and Maillard reaction products, whereas others are destroyed in the heat treatment. Some studies have been undertaken relating to the volatiles characterization of tomato sauces being the work of Bendini et al. (2017) that which provides more information about commercial tomato sauces [8].

The volatiles extraction step is a complex procedure affected by different factors such as extraction temperature, stirring, sample weight, and extraction time, among others. Thus, to obtain complete profiles of volatiles it is common to apply multivariate statistical methodologies and experimental design. Response surface methodology (RSM) is an effective technique which is widely used for optimizing the process parameters with the evaluation of the independent variables and their interactions on the dependent variables with the reduced number of trials [9]. In particular, Box–Behnken designs (BBD) are nearly rotatable second-order designs based on three-level incomplete factorial designs [10]. BBD avoids experiments performed under the highest or lowest levels, which is why it has been successfully applied to optimize the extraction process of volatile compounds by headspace solid-phase microextraction (HS-SPME) in different food matrices such as roasted sweet potato [11], blackberry [12] and so on. Nevertheless, the optimization of the HS-SPME extraction procedure of volatile compounds from *sofritos* done with different ingredients has not been reported in the literature before.

Therefore, the aim of the present work was to compare the total antioxidant capacity and the volatile organic profile of different formulations of homemade *sofrito* cooked in the same control conditions but changing one of the ingredients in order to know if the addition of herbs or the elimination of one the characteristic ingredients affects notably on the antioxidant capacity and on the volatile profile. To achieve this purpose, optimization of the volatile’s extraction process was undertaken.

## 2. Materials and Methods 

### 2.1. Materials

Sodium carbonate, sodium chloride, glacial acetic acid, ferric chloride, potassium persulfate, methanol (high-performance liquid chromatography (HPLC) grade), n-hexane (99%, gas chromatography (GC) grade) were obtained from Panreac (Barcelona, Spain). Gallic acid, (±)-6-Hydroxy-2,5,7,8-tetramethylchromane-2-carboxylic acid (TROLOX), Folin–Ciocalteu reagent, 2,2-azinobis (3-ethylbenzothiazoline-6-sulfonic acid) diammonium salt (ABTS); 2,2-diphenyl-1-picrylhydrazyl (DPPH), 2,4,6-tris(2-pyridyl) S-Triazine (TPTZ), hexanal, trans-2-heptenal, octanal and trans-2-decenal volatiles standards were acquired from Sigma–Aldrich Inc. (St. Louis, MO, USA). Spring Garlic (Peregrin, Almeria, Spain), Picual virgin olive oil (Tierras de Tavara, Jaen, Spain), onions and crusted pear red tomato jar were obtained in a supermarket in Alicante (Spain) whereas dried thymus and Rosmarinus were acquired from the company Carmencita (Alicante, Spain).

### 2.2. Preparation of Tomato Sofrito and Dry-Matter Determination

Table 1 shows the compositions of tomato *sofrito* elaborated in this work. Samples were prepared by using the food processor Thermomix TM5 (Vorwerk, Thermomix, Madrid). Tomato *sofrito* number 1 (CS1) was the original standard recipe. Thus, extra virgin olive oil was heated at Varoma temperature (120 ℃) and stirring at speed 1 (40 rpm). Then, chopped garlic was added and cooked for 5 min at the same temperature under stirring (40 rpm). After that, diced onions and canned tomato were added and cooked for 20 min at Varoma temperature under stirring (40 rpm). Finally, the mixture was chopped during 6 s under stirring (4130 rpm). Based on this original recipe and modifying only one ingredient each time, five different variants of *sofrito* were produced (CS1 to CS6) (Table 1). Furthermore, three more samples, based on the mixture of the raw ingredients (S1), raw onion (S8) and raw garlic (S9) were homogenized in the Thermomix at 4130 rpm during 10 s without heating. The yield factor was also calculated for each type of *sofrito* [13].

The dry matter of samples was measured gravimetrically, drying the samples at 104 ℃ in an oven Selecta 2000200 (Barcelona, Spain) during 24 h or until constant weight.

### 2.3. Preparation of Antioxidant Extracts

A slightly modified method described previously was used [14]. Briefly, 1.0 ± 0.1 g of homogenized sauce sample was weighed in a 10 mL polypropylene tube and then 4 mL of a methanol: water (80:20 *v/v*) solution was added. The mixture was homogenized for 1 min in a Vortex shaker and centrifuged for 10 min at 5000 rpm. The supernatant was collected and passed to another 10 mL tube with a Pasteur pipette. The extraction process was repeated twice. The two supernatants were pooled and stored in the fridge at −18 ℃ until analysis. All samples were extracted in triplicate.

### 2.4. Antioxidant In Vitro Activity

Four complementary methods were used to determine the antioxidant activity of methanolic extracts. FRAP, DPPH and total phenols by the Folin–Citocalteau electron transfer methods and ABTS a method based on hydrogen atom transfer [15]. The standard curve was prepared using TROLOX as standard in the range of concentration from 0 to 500 µmol L^−1^ for FRAP, DPPH and ABTS. Results were expressed as µmol equivalents of TROLOX per 100 g of *sofrito*.

Moreover, gallic acid was selected as the reference standard in the range from 0 to 500 mg L^−1^ for the total phenols using Folin–Citocalteau Method. Results were expressed as mg gallic acid per 100 g of sample. All tests were done in triplicate.

#### 2.4.1. DPPH (2,2-Diphenyl-1-Picrylhydrazyl) Method

An aliquot of 100 µL of the *sofrito* extracts (0–10 mg mL^−1^) was added to 2.5 mL of 24 mg L^−1^ methanol solution of DPPH and the kinetics of the reaction was followed monitoring the absorbance spectrophotometrically at 517 nm until the signal reached a stable value. For the studied samples, 90 min was selected as steady reaction time. Subsequently, all samples were measured after 90 min of incubation in the dark at room temperature (25 ± 2 ℃) with the DPPH radical at 517 nm using a spectrophotometer (Biomate-3, Thermospectronic, Mobile, AL, USA) [16].

#### 2.4.2. ABTS (Diammonium Salt) Method

The ABTS scavenging activity was measured according to previous work with some modifications [17]. The ABTS radical cations were prepared by mixing 25 mL of 7 mM ABTS solution with 88 µL potassium persulfate (140 mM) solution. The solution obtained was incubated in the dark for 16 h. The ABTS solution was then diluted with ethanol 96% conveniently until obtaining an absorbance at 734 nm of 0.70 ± 0.02. Then, 100 µL of extracts (0–10 mg mL^−1^) were combined with 3 mL of the ABTS solution and homogenized with a vortex. The reaction mixture was incubated at room temperature (25 ± 2 ℃) for 10 min and then the absorbance was measured at 734 nm against a blank (ABTS solution with 100 µL of methanol: water (80:20)) using a spectrophotometer (Biomate-3, Thermospectronic, Mobile, AL, USA).

#### 2.4.3. FRAP (Ferric-Reducing Antioxidant Power) Method

The reduction capacity of the ferric cation of methanolic extracts was assessed according to Benzie and Strain (1996) method with several modifications [18]. The FRAP reagent was prepared every working day by mixing a sodium acetate buffer of 300 mM at a pH of 3.5 with a solution of 10 mM of TPTZ and a 20 mM FeCl_3_·3H_2_O solution in a volume relation of 10:1:1. An extract aliquot of 100 µL was mixed with 3 mL of FRAP reagent and incubated for 30 min in the dark at 25 ± 2 ℃. Measurements were performed with a spectrophotometer at 593 nm.

#### 2.4.4. Total Polyphenols Content (TPC)

The TPC assay was adapted from a previously published work [19]. 200 µL of the methanolic extract was mixed with 100 µL of Folin–Citocalteu’s reagent (0.5 N) and 900 µL of a 7% sodium carbonate solution. The mixture was homogenized with a Vortex stirrer for 10 s followed by an incubation period in a water bath at 50 °C for 30 min. The absorbance was measured at 760 nm in a spectrophotometer (Biomate-3, Thermospectronic, Mobile, AL, USA) using deionized water as blank.

### 2.5. Analysis of Volatile Compounds by Headspace Solid-Phase Microextraction-Gas Chromatography Mass Spectrometry (HS-SPME-GC-MS)

#### 2.5.1. HS-SPME Sampling

The required amount of sample was weighted out directly into the 20 mL amber vial, 2 mL of distilled water (with or without NaCl addition) and a micro-stirring bar were added to the vial. The vial was closed with an aluminium crimp cap provided with a needle-pierceable polytetrafluoroethylene/silicone septum. The SPME fibre used was divinylbenzene/carboxen/polydimethylsiloxane (DVB/CAR/PDMS) 50/30 mm, StableFlex, 1 cm long, mounted to an SPME manual holder assembly (Supelco, Bellefonte, PA). This fiber has extensibility shown to be suitable for the volatile extraction compounds from tomato sauce preserved in different packaging materials (glass, tin and multilayer plastic containers) [20], Italian and Spanish ready-to-eat tomato sauces [8] and other vegetable matrices used as part of *sofrito* such as onion [21] and extra-virgin olive oil [22].

The sample vial was then placed over a thermostatic water bath and it was stirred at 500 rpm. After 10 min of sample equilibration, the SPME needle was exposed to the volatile’s compounds in the headspace of the vial by inserting it into the vial through the septum. After the required extraction time, the SPME needle was taken out of the tube and the fibre was immediately desorbed into the GC-MS injection port at 250 °C for 10 min (splitless mode). All tests were carried out in triplicate. Blank runs were carried out before sample analysis until no contamination on the fibre was found due to memory effects

#### 2.5.2. Box–Behnken Experimental Design (BBD)

Table S1 in Supplementary data.

Four independent factors were considered for HS-SPME optimization with a BBD model: sample weight (A = 0.50, 1.75, 3.00 g), extraction temperature (B = 35.0, 47.5, 60.0 ℃), extraction time (C = 10, 35, 60 min), addition of 2 mL of NaCl in distilled water into the vial prepared at three different concentrations (D = 0, 1, 2 M) (see Table S1). The CS1 was selected for the optimization of HS-SPME conditions as it contains the basics ingredients of this sauce. A total of 29 experiments (3-level design including a 24 subset of the runs in the full three-level factorial and 5 centre points to estimate the experimental error) were carried out in randomized order. The quadratic model that relate the responses of the system (dependent variable) in the function of the studied factors (independent variables) is shown in Equation (1).

Y = β0 + Σβi Xi + Σβi,iX2 + ΣΣβi,j XiXj B2 + ε(1)

where Y is the predicted response, X represents the variables of the system, i an j are design variables, β0 is a constant, βi are the first-order coefficients, βi,i the quadratic coefficients for ith factors, βi,j the coefficients for the interaction of factors i and j and ε is the error.

Optimization was performed to obtain the maximum extraction efficiency in the sum of areas obtained for hexanal, trans-2-decenal, octanal and trans-2-heptenal. These volatile compounds were selected because they are aldehydes typically detected as markers of fresh tomato flavour (the main ingredient of the *sofrito* sauce) and, also, they could be modified during the thermal processing [8,20,23]. The experimental conditions that maximized the response were obtained from the fitted model using StatGraphics Centurion XV software (Statistical Graphics Corporation, Rockville, MD, USA).

#### 2.5.3. Gas Chromatography-Mass Spectrometry (GC–MS) Analysis

Analysis of volatiles was carried out using an Agilent 6890N GC coupled to a 5973N MS (Agilent Technologies, Palo Alto, CA) operating in electron ionization mode (EI 70 eV). Ion source and GC-MS transfer line temperatures were 230 and 280 °C, respectively. A DB-624 column, 30 m × 0.25 mm × 0.14 mm (Agilent Technologies, Palo Alto, CA) was used and it was programmed from 50 (hold 3 min) to 250 °C at 10 °C min^−1^ (hold 12 min). Helium was used as the carrier gas (1 mL min^−1^). Peak identification was based on the comparison of mass spectrum data with spectra in full scan mode (m/z 30–550) present in the Wiley library considering the volatile compounds that matched equal or more than 90% similarity. Semiquantitative data was applied and expressed as the percentage composition of each peak with respect to the total volatile peak area in S1 [12]. Hexanal, trans-2-decenal, octanal and trans-2-heptenal (98%, Sigma–Aldrich, St. Louis, MO) were employed for the validation of the quantification method using calibration curves at six concentrations levels, in triplicate, prepared using distilled water as a solvent. Three replicates for each sample were performed.

#### 2.5.4. Volatiles Quantification Validation

The analytical method used for volatiles quantification was validated in terms of linearity, limits of detection (LOD), quantitation (LOQ) [24] and precision in terms of the intra- and inter-day repeatability. The peak area ratio which is the ratio of the total chromatographic peak area of the analytes was plotted against the concentration of analytes. LOQ and LOD values were determined by using regression parameters from the calibration curves at five concentration levels, in triplicate (3 Sa/b and 10 Sa/b, respectively; where Sa is the standard deviation of the residues and b is the slope). The repeatability is expressed as the relative standard deviation (RSD) of peak areas of triplicates. The intra-day precision (*n* = 3) was estimated by performing three extractions under optimal conditions in a single day, and inter-day precision (*n* = 9) was estimated based on three extractions under optimal conditions per day over three consecutive days.

### 2.6. Statistical Analysis

Statistical analysis of experimental data was performed with SPSS commercial software (Version 15.0, Chicago, IL). A one-way analysis of variance (ANOVA) was carried out. Differences between means were assessed based on confidence intervals using the Tukey test at a confidence level of 95% (*p* < 0.05). Cluster analysis was carried out by applying the Ward distance as a method for agglomeration and the square of Euclidean distance as a criterion of similarity.

## 3. Results and Discussion

### 3.1. Dry Matter

The dry matter (%) is shown in Table 1. The original recipe, ie. CS1 was significantly modified by changing one of the ingredients. As expected, the cooking process reduced the final moisture content since the raw ingredients corresponding to S1 presented a dry matter of 17.3 ± 0.2% whereas, in CS1, the value decreased to 27.4 ± 0.2% [7]. Cooked *sofrito* 6 registered the highest dry matter (33.2 ± 0.4%). This fact can be explained because it is the only sauce that does not contain onion, this ingredient being that with the higher water content in the raw form reporting a value of 8.6 ± 0.2%. On the other hand, the yield factors (YF) of the *sofritos* were calculated [13] and the values for CS1-CS5 were very similar around 0.54–0.56, meanwhile, CS6 showed a YF around 0.50 ± 0.2. This means that this sample should be more concentrated in components than in the rest of the samples.

### 3.2. Analysis of Total Antioxidant Capacity

The values of total phenol content (TPC) were found to range from 86 to 137 mg GAE 100 g^−1^ wet sofrito (WS). Comparison of total polyphenol data with other tomato sauces is difficult as the results depend on several parameters such as the proportion of the ingredients on the recipe, the cooking technique, processing time, dry or fresh sample and, also on the extraction of the sample. According to the phenol-explorer database on polyphenol content in foods, raw tomato, onion, olive oil, garlic, rosemary and thymus have average values of 45.06, 60.15, 18.31, 87.04, 2518 and 1815 mg gallic acid equivalent (GAE) 100 g^−1^ fresh weight, respectively [25]. The TPC levels obtained for raw onion and garlic were 86.0 and 110.4 mg GAE 100 g^−1^ fresh weight, respectively. So the values obtained were in accordance with those extracted from phenol-database.

The mixture of the fresh ingredients conforming S1 presented an average in TPC of 86 ± 2 mg GAE 100 g^−1^ fresh sample meanwhile the cooked version and its recipe modifications showed a TPC of 97 ± 10, 120 ± 8, 114 ± 8, 137 ± 12, 93 ± 5 and 114 ± 9 mg GAE 100 g^−1^ WS for CS1, CS2, CS3, CS4, CS5 and CS6, respectively. It seems that the cooking process slightly increased the TPC of the *sofrito*. These results are in accordance with other authors that have reported the benefits of thermal processing of tomato sauces, including inactivation of enzymes, prolongation of shelf-life and improving the digestibility and bioavailability of nutrients including antioxidants [7,26]. Consistent with these results, Tomas et al. reported that heat treatment may result in changes in the extractability of phenolics and other compounds due to the disruption of the plant cell wall with the easier release of bound polyphenolic compounds [7]. The addition of thyme, rosemary or double the quantity of garlic increased significatively the TPC respect to CS1 which is logical as those ingredients are rich in polyphenols. The elimination of garlic in sample CS5 did not have a significant effect on TPC probably due to the low proportion of garlic respect to the rest of ingredients. Finally, the removal of onion conducted to a significant higher TPC in CS6 compared to CS1. This could be explained for the differences in dry matter of the final sauce as was shown in Table 1 and the different proportion of ingredients compared to the rest of the *sofrito*s.

**Figure 1:**
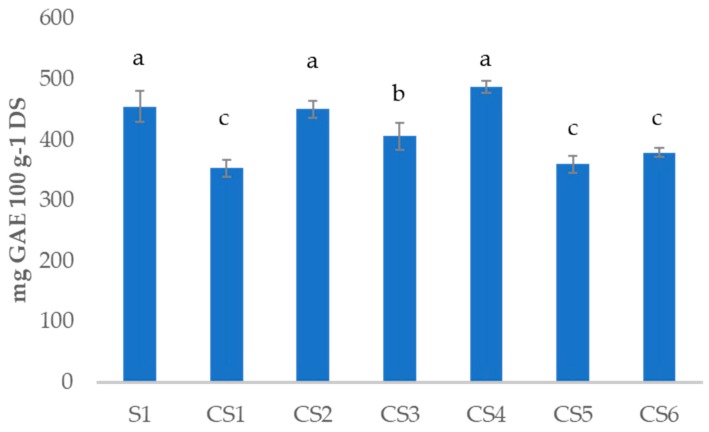
Total phenol content (TPC) of all the sample analyzed expressed in mg gallic acid 100 g^−1^ dried sample (DS). Different superscripts indicate statistically significant different values (*p* < 0.05). raw *sofrito* 1 (S1); cooked *sofrito* 1–6 (CS1–6).

In order to evaluate the possible influence of different water content among the samples Figure 1 shows the TPC of the samples expressed in mg of gallic acid 100 g^−1^ of dry sample (DS). The comparison of TPC on the dried basis shows that the raw sample (S1) present significant higher content than the cooked one CS1 and the samples CS5, CS6 and CS3. Therefore the final content will depend on the probable destruction or transformation and isomerization of part of the polyphenols during the cooking process as observed previously [27], and also the liberation of polyphenols from the vegetable cells [7]. The addition of double garlic and rosemary added additional polyphenols to the recipe resulting in a non-significant TPC compared to the raw S1.

**Figure 2:**
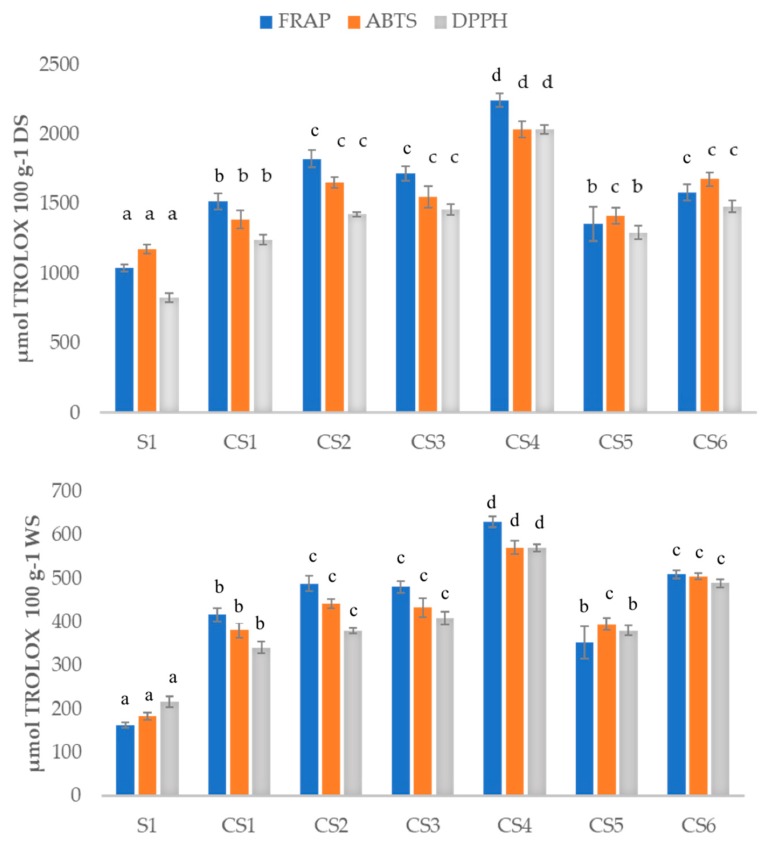
Antioxidant capacity calculated with ferric-reducing antioxidant power (FRAP), diammonium salt (ABTS) and 2,2-diphenyl-1-picrylhydrazyl (DPPH) methods for all the samples analyzed expressed in µmol equivalents of TROLOX 100 g^−1^ dried (DS) or wet sample (WS). raw *sofrito* 1 (S1); cooked *sofrito* 1–6 (CS1–6). Different superscripts in the results of the same method indicate statistically significant different values (*p* < 0.05).

In relation to the antioxidant capacity of the samples, this was compared using the FRAP, ABTS and DPPH assays as the use of a single antioxidant assay is not completely representative considering that different reactive species and mechanisms are involved in oxidative stress in vivo [28]. From the results obtained shown in Figure 2, it can be concluded that home processing of the *sofrito* samples resulted in a significant relative increase in antioxidant capacity compared to the raw sample S1 independently of the water content. This can be appreciated in Figure 2 were the antioxidant capacity was expressed both as µmol TROLOX per 100 g^−1^ WS and per 100 g^−1^ DS. To note the different behaviour of antioxidant capacity and TPC on a dry basis, as for the antioxidant capacity the S1 value is lower than for the cooked *sofrito*s. This can be because antioxidant capacity does not depend only on the polyphenol content but also on other species such as carotenoids, tocopherols, phytosterols, metals, etc. [29]. Some of these compounds are not water soluble. In addition, the formation of Maillard products due to heating processing may also modify some of the species responsible for the antioxidant properties.

Regarding the antioxidant capacity of specific *sofrito* samples (Figure 2), the higher antioxidant activity content evaluated was obtained again for CS4, which contains 1 g of rosemary. Adding aromatic herbs, such as thyme and rosemary increased significantly the antioxidant capacity as reported in Figure 2 in relation to S1. This is a logical result as these ingredients are very rich in polyphenols and other antioxidants as mentioned below [30,31]. Additionally, the elimination of an important ingredient such as onion in CS6 also affected the antioxidant capacity. In this case, its higher value compared to CS1 must be related to the higher proportion in the recipe of carotenoids, vitamin E and vitamin C from tomatoes as onion lacks those vitamins (Base de datos Española de composición de alimentos, BEDCA database) [32]. Additionally, an improvement in the bioaccessibility of Z-carotenoids when tomato is cooked with olive oil has also been documented before [2]. Furthermore, the addition of a double quantity of cooked garlic in CS2 led to a significantly higher value of antioxidant capacity compared to CS1. This difference could be attributed to the presence of the ajoene and vinyldithiins compounds, which present quite elevated TPC values [33]. These results showed the same tendency among the three methods assayed giving a suitable correlation among them. The significant differences were calculated applying one-way ANOVA. Different letters in Figure 2 show significant differences at *p* < 0.05.

In relation to the cooked *sofrito* samples, the values obtained in this work were similar to those obtained in baked and fried tomatoes by other authors [34] and higher in comparison with some commercial ones [35]. The differences could be attributed to the use of seed oil instead of extra virgin olive oil, the cooking temperature, and the rough processing technologies such as sterilization used in the commercial preparation of *sofrito* samples as it has been reported previously by other authors [35].

In conclusion, the TPC in all the cooked samples showed a slightly lower value than in the raw sample in relation to dry matter. As the yield factor was around 0.50–0.56 some phenols must have been destroyed during the processing of the *sofrito* as observed somewhere else [16]. On the contrary, the other three antioxidant capacity methods showed higher values in cooked samples than in the raw one. This is due to the presence of antioxidant species different to polyphenols like carotenes, and metals, that resist better the cooking process.

### 3.3. Experimental Design for Optimization of HS-SPME Procedure for Volatiles Extraction

The optimization procedure was carried out to obtain the optimal levels of the four studied independent variables (A: sample weight, B: extraction temperature, C: extraction time; D: NaCl addition), their quadratic effects and interactions leading to the highest headspace concentrations of hexanal, trans-2-heptenal, octanal and trans-2-decenal. Numerical optimization was also carried out using the RSM to determine the exact optimum levels of the independent variables leading to a desirable response goal. The Pareto chart estimates the statistical significance of the factors and interactions between them that had the greatest effect on the response (Figure S1).

Among the studied factors, five had a significant effect on the response. The extraction temperature (B) had the greatest influence on the studied response, showing a positive effect. Also, the extraction time (C) and the interaction temperature and time (BC) had a significant and positive effect, followed by the tomato sauce weight (A) factor and its quadratic interaction (AA) with a negative effect. The rest of the investigated parameters had no significant impact on the studied responses. These results could be explained because of the cooking processing on the raw cellular structure of the *sofrito* ingredients. Higher extraction temperatures and time allow the disruption of vegetable cell walls and, consequently, the inner contents of the cells become more accessible, as for example to volatiles compounds [3]. On the other hand, the transport of analytes from the matrix into the coating begins as soon as the coated fibre has been placed in contact with the sample. However, if the diffusion coefficient of the target compounds is low, the analyte remained at the interface and adsorption of target compounds decreased. This fact explained the negative effect of the quadratic interaction of *sofrito* weight since the formation of high analyte concentrations saturates the closed vial headspace, increasing the competition of target and interference compounds to be absorbed in the fibre coating and, consequently, their extraction [36].

By applying multiple regression analysis on the experimental data, the mathematical model representing the studied response as a function of the independent variables within the region under investigation were expressed by Equation (2).

*Y* = 2.09012 × 10^−7^ − 3.49115 × 10^−6^ × A + 1.02543 × 10^−7^ × B + 9.37307 × 10^−6^ × C − 1.05587 × 10^−6^ × D − 4.58137 × 10^−6^ × A^2^ − 5.61886 × 10^−6^ × AB − 2.66642 × 10^−6^ × AC − 867317 × AD + 1.34314 × 10^−6^ × B^2^ + 7.04733 × 10^−6^ × BC − 3.23108 × 10^−6^ × BD − 2.61914 × 10^−6^ × C^2^ − 1.02289 × 10^−6^ × CD − 1.3836 × 10^−6^ × D^2^ (2)

The test was performed by comparing the variability of the current model residuals to the variability between observations at replicate settings of the factors. The R-squared statistic indicates that the model as fitted explains 87.29% of the variability in the response. This value indicated a relatively high degree of correlation between the actual data and predicted values, confirming that the model obtained could be used to predict the studied responses. In contrast with reported literature, this optimized HS-SPME procedure can reduce the sample weight required during the extraction from 3 g [8] and 1.5 g [20] to 0.5 g tomato sauce, whereas the extraction time (60 min) was similar to reported ones. However, extraction temperature is slightly higher in this application in contrast to other non-experimental optimized methods which reported the extraction step at 40 ℃.

In the aforementioned literature, different volatiles have been reported (including alcohols, aldehydes, esters, furans, and ketones) in the volatile fraction of tomato sauces but, the four target compounds in this study, belong to the aldehydes chemical class and encompassed a range of molecular weights with long chains length from six to ten carbon atoms. Thus, the optimized HS-SPME procedure obtained may require a higher extraction temperature [24]. This condition could be an advantage for the extraction of the rest sofrito volatiles since several authors have suggested that heating provides energy for analyte molecules to overcome energy barriers that tie them to the matrix. Increasing temperature will enhance the mass transfer process, increasing the vapour pressure of the analytes, and thereby facilitating the release of analytes into the headspace [24,37].

### 3.4. Volatile Compounds Profile Identification

**Figure 3:**
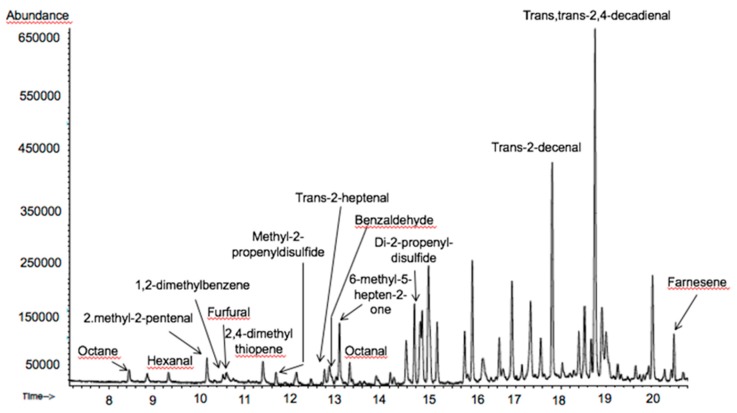
Total ion current (TIC) chromatogram obtained for CS1 used for the optimization procedure by the Box–Behnken designs (BBD) and main volatile compounds identification.

Figure 3 shows the total ion current (TIC) chromatogram obtained for *sofrito* 1 used for the optimization procedure by the BBD. Peak identification was based on the comparison of mass spectrum data with spectra in full scan mode (m/z 30–550) present in the Wiley library that matched equal or more than 90% similarity.

A total of 41 volatile compounds were identified among all the samples analyzed (Table 2). In S1 14 volatile organic compounds (VOCs) were identified meanwhile two more VOCs were encountered in CS1. Some of the volatiles were only detected in the raw sample, those were: 1-octanol (1,3%), 2-hexenal (0.67%), di-2-propenyltrisulfide (3.1%) and 3,4-dihydro-3-vinyl-1,2-ditin (26.3%). The last two compounds are related to garlic and/or onion and represent nearly 30% of the total peak area. It is known that thiosulfonates are very unstable during cooking procedures and they usually rearrange to poly-sulfurs that still have biological activity [38].

### 3.5. Validation of the Quantification Method for the Volatile Compounds

The GC-MS method was optimized and validated for this application the validation parameters are shown in Table 3. Acceptable linearities were obtained using a set of calibration curves prepared with six standards diluted using distilled water in the range of concentration shown in Table 2. Additionally, satisfactory reproducibility and repeatability of the method were observed. Furthermore, LOD were between 0.89–1.55 µg Kg^−1^, meanwhile for trans-2-decenal was a little bit higher near 20 µg Kg^−1^., these LOD are very similar to a previous work in which multiple HS-SPME was employed.

In the present study, the response factor for the four aldehydes quantified was significantly different, the slope of the curve being higher as the length of the chain of the molecule increased. These means that for a quantitative study standard would be necessary for all volatiles. Alternatively, a semiquantitative determination will be done based on the peak are percentage.

For the semiquantitative determination, per cent peak area respect to S1 total area was calculated in order to be able to compare among samples. The relative areas are shown in Table 3. Important differences in peak area were observed when comparing the VOC presented in S1 with the cooked *sofritos* (CS). Some compounds detected in the samples were related to tomato and its cooking process. Hexanal is important in the fresh tomato flavour contributing to the green aroma. This aldehyde together with [21] 2-methyl-2-pentanal and benzaldehyde diminished in the cooking process [23]. Others like trans-2-heptenal, 6-methyl-5-hepten-2-one did not change significantly the percentage in all the samples [8,23]. By contrast, other compounds were not detected in S1 and were quantifiable in the cooked *sofritos*. Those compounds were three aldehydes, trans, trans-2,4-decadienal represents (13.6–1.9%) and trans-2-decenal in important proportion (7.5%–5.4%) and octanal in a lower proportion. Unsaturated aldehydes are usually derived from a linoleic acid breakdown pathway [23], which is reasonable after the cooking process [39]. No significant differences were found among all cooked *sofrito*s in trans-2-decenal and octanal. Meanwhile trans, trans-2,4-decadienal was significantly different from S5 and S6 to S1–S4 (Table 2). Moreover, octane and 1,2-dimethyl benzene were also detected in the cooked samples probably related to the Maillard reaction [40].

On the other hand, several studies have reported that 2-methyl-2-pentenal, methyl-2-propenyldisulfide, 2,4-dimethylthiopene, di-2-propenyldisulfide, dipropyl disulfide, trans-propenylpropyldisulfide, di-2-propenyltrisulfide and 3,4-dihydro-3-vinyl-1,2-dithiin are volatile compounds linked with the presence of garlic and/or onion as ingredients in the tomato sauce [21,33,40]. In this sense, di-2-propenyldisulfide, methyl-2-propenyldisulfide, were present in CS1 and CS2 but not in CS5 which contains no garlic. In contrast, alpha-farnesene, 2,4-dimethyl-thiophene, 2-methyl-2-pentenal could be linked with the presence of onion since it was not identified or in lower quantity in CS5 formulated without garlic. Some of these compounds, specially disulfide and trisulfide derivates are known to have antioxidant properties [29]. Regarding the olive oil ingredients, four main volatiles were identified (2,4-heptadienal, 1,2-dimethyl-benzene, benzeneacetaldehyde and 1-octanol) [41].

Additionally, it was observed that the total peak area detected in the raw sample was higher than the area obtained in cooked samples but for CS3 and CS4. This result indicates that during de cooking process part of the VOCs escape to the air [42]. In the case of CS3 and CS4 the addition of rosemary and thymus increased considerably the number of VOCs as can be observed in Table 3. The volatile profiles of CS3 and CS4 were noticeable due to the presence of seven common compounds that are linked to the presence of thymus and rosemary in each formulation (linalool, 4-terpineol, borneol L, α-terpineol, 1-methyl-4-(1-methylethyl)-benzene, β-caryophyllene and delta-cadinene) [43,44]. However thymol and 2-methyl-5-(1-methylethyl)-phenol were only detected in *sofrito* number 3 linked with thyme ingredient [44] whereas 8 additional compounds were characterized in *sofrito* number 4 (camphene, myrcene, eucalyptol, camphor, α-copaene, α-humulne and α-amorphene) that are expected to be extracted from rosemary. Most of these compounds are phenolic terpenes with known antioxidant properties [29].

**Figure 4:**
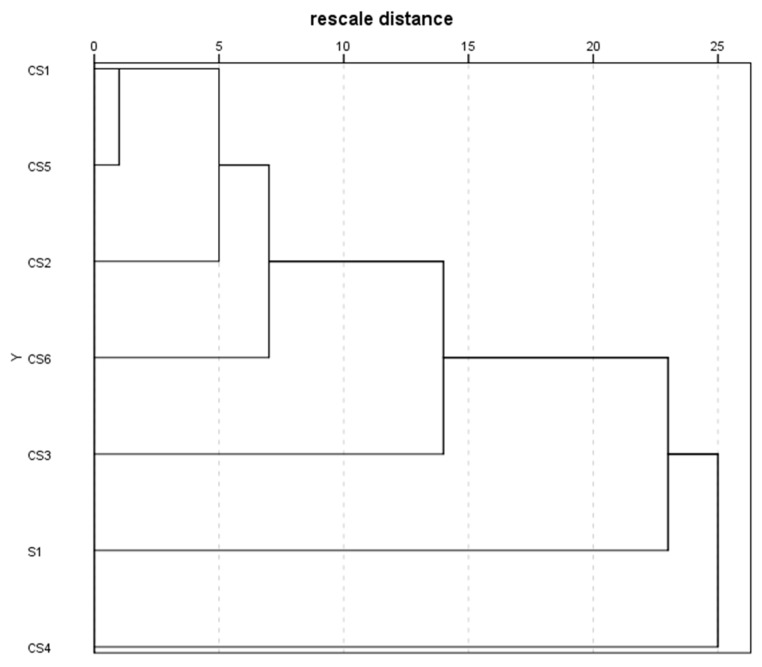
Dendrogram obtained after applying hierarchical cluster analysis to the volatile’s percentages data.

As some of the volatiles identified in this study are terpenoids or phenols with antioxidant capacity a cluster analysis was applied to the volatile data trying to classify the samples based on their dissimilarities in VOCs. The dendrogram obtained is shown in Figure 4 and shows three groups at a rescaled distance of 15. One of the groups was constituted by S1 and the other one by CS4, and a third group was formed by the samples CS1 and CS5 being the most similar, followed by CS2 and CS6. These dissimilarities are the same found before when the antioxidant capacity was considered. CS4 is the sample with the higher TPC and antioxidant capacity which agrees with the sample with the greater amount of antioxidant volatiles such as α-terpineol, camphor, myrcene and α-copaene. Additionally, S1 presents sulfur-containing compounds (di-2-propenyl disulfide and di-2-propenyl trisulfide) that have antioxidative properties [45] and are not present in the cooked samples which make this sample different to the others. Finally, CS3 is the most different sample in the third group. CS3 contains some volatiles such as thymol and 2-methyl-5-1-methyl ethyl-phenol with antioxidant properties not present in the rest of the samples.

## 4. Conclusions

In general, the cooking process improved the release and the concentration from the food matrix of interesting bioactive compounds in *sofritos* but also their volatilization. The addition of one or other type of herbs has an important effect on the final antioxidant capacity and also in the volatile profile event, although these ingredients are used in very low proportion in the recipe. This phenomenon is positive from a nutritional point of view as cooking can modify notably the flavour and healthy properties of a dish but also can help to increase the shelf-life of these dishes as the antioxidant capacity is enhanced. In this study CS4 with rosemary seemed to be a more resistant sample to degradation due to its higher TPC and antioxidant activity. This result correlates with the highest quantity in VOCs presented in this sample some of them identified as potent antioxidant compounds [46]. Even in a lower proportion, the addition of thyme or greater quantity of garlic is also beneficial from an antioxidant point of view [47]. In this sense additional studies will be done in the future in which the cooking technique employed is evaluated as this is variable and was not evaluated in this work. Depending on the dry matter of the sauce, the TPC values can vary substantially being very important to consider when the experimental data are compared with published data. In our case, the results have been expressed in both ways (dry and wet matter) but nutritionally speaking the valid results are those expressed as wet matter as that is the way we consume the product. Additionally, some of the VOC could be used to explore if one ingredient was added to a preparation such as terpineol (rosemary); and thymol (thymus).

## Figures and Tables

**Table 1:** Recipes composition (g), dry matter content (%) and yield factor (YF) of the different *sofrito* samples.

Recipe *	Tomato	Onion	Garlic	Olive Oil	Salt	Thyme	Rosemary	Dry Matter % **	YF
CS1	400	150	15	50	2			27.4 ± 0.2a	0.54 ± 0.1
CS2	400	150	30	50	2			26.7 ± 0.1b	0.56 ± 0.2
CS3	400	150	15	50	2	1		28.0 ± 0.1c	0.54 ± 0.1
CS4	400	150	15	50	2		1	27.9 ± 0.1c	0.56 ± 0.1
CS5	400	150	0	50	2			26.0 ± 0.1d	0.55 ± 0.1
CS6	400	0	15	50	2			33.2 ± 0.4e	0.50 ± 0.2
S1	400	150	15	50	2			17.3 ± 0.2f	
Raw onion								8.6 ± 0.2g	
Raw garlic								33.8 ± 0.2e	

* Cooked sofrito (CS); raw sofrito (S1); ** Different letters in the columns represent statistically significant differences (*p* < 0.05). Dry matter content expressed as average quantities (%) ± standard deviation of three replicates for each sample (*n* = 3).

**Table 2:** Validation parameters for the headspace solid-phase microextraction-gas chromatography mass spectrometry (HS-SPME-GM-MS) optimized method.

Validation Parameter	Hexanal	Trans-2-Heptenal	Octanal	Trans-2-Decenal
Linear range (mg Kg^−1^)	0.001–1.096	0.001–1.760	0.002–0.880	0.004–0.073
R^2^ value	0.998	0.997	0.998	0.990
Limits of detection (LOD) (µg Kg^−1^)	0.89 ± 0.06	1.03 ± 0.53	1.55 ± 0.07	18.48 ± 0.65
Limits of quantitation (LOQ) (µg Kg^−1^)	2.97 ± 0.20	5.76 ± 0.78	5.15 ± 0.24	61.59 ± 2.18
Intra-day repeatability *	1.9	4.2	1.4	3.5
Inter-day repeatability *	6.7	4.9	6.5	7.9

* Intra-day and inter-day repeatability (peak area relative standard deviation (RSD) %).

**Table 3:** Percentage of peak area of the total volatile composition of tomato sauces obtained in the HS-SPME/GC–MS analysis directly calculated from total ion current (TIC) (Mean ± standard deviation (SD), *n* = 3) and retention times.

Compound	Rt (min)	S1	CS1	CS2	CS3	CS4	CS5	CS6
trans-2-heptenal	12.77	0.66 ± 0.07a	0.83 ± 0.36b	0.73 ± 0.14b	0.75 ± 017b	0.88 ± 0.36b	0.88 ± 0.11b	1.11 ± 0.21b
2-hexenal	10.75	0.67 ± 0.04	Nd	Nd	Nd	Nd	Nd	Nd
1-octanol	14.58	1.26 ± 0.21	Nd	Nd	Nd	Nd	Nd	Nd
2,4-dimehyl-thiophene	11.41	1.34 ± 0.31a	1.64 ± 0.14a	1.36 ± 0.14a	1.26 ± 0.21a	1.43 ± 0.33a	1.76 ± 0.25a	0.28 ± 0.01b
furfural	10.59	1.36 ± 0.23	0.84 ± 0.11	1.02 ± 0.21	1,34 ±0.14	1.26 ± 27	1.35 ± 0.12	Nd
hexanal	9.32	1.70 ± 0.32a	0.53 ± 0.06b	0.60 ±0.11b	0.51 ± 0.05b	0.45 ± 0.25b	0.70 ± 0.10b	0.89 ± 0.12c
benzaldehyde	13.01	1.70 ± 0.42a	0.44 ± 08b	0.39 ± 0.10b	0.44 ± 0.07b	0.42 ± 0.07b	0.33 ± 0.03b	0.38 ± 0.04b
6-methyl-5-hepten-2-one	13.10	1.82 ± 0.45	2.16 ± 0.84	3.55 ± 0.85	2.21 ± 0.74	3.04 ± 0.73	3.24 ± 0.40	3.63 ± 0.35
alpha-farnesene	20.51	1.99 ± 0.56	0.89 ± 0.18	0.71 ± 0.33	1.03 ± 0.19	1.08 ± 0.54	0.47 ± 0.20	Nd
di-2-propenyltrisulfide	18.38	3.05 ± 0.46	Nd	Nd	Nd	Nd	Nd	Nd
methyl-2-propenyldisulfide	11.70	3.68 ± 0.65a	0.50 ± 0.06b	0.85 ± 0.18b	0.65 ± 0.15b	0.64 ± 0.28b	Nd	0.22 ±0.02c
2-methyl-2-pentenal	10.16	5.40 ± 0.70a	2.21 ± 0.69b	1.99 ± 0.16b	1.49 ± 0.38b	1.18 ± 0.40b	1.52 ± 0.30b	0.34 ± 0.01c
3,4-dihydro-3-vinyl-1,2-dithiin	16.92	26.34 ± 3.13	Nd	Nd	Nd	Nd	Nd	Nd
di-2-propenyldisulfide	14.76	47.12 ± 5.20a	2.84 ± 0.57b	16.76 ± 1.30c	4.94 ± 0.91b	2.99 ± 1.31b	0.58 ± 0.10d	6.36 ± 0.39b
1,2-dimethyl-benzene	10.52	Nd	0.55 ± 0.26	0.33 ± 0.07	0.41 ± 0.08	0.47 ± 0.18	0.60 ± 0.18	Nd
benzeneacetaldehyde	14.57	Nd	2.27 ± 0.48	1.14 ± 0.23	2.69 ± 0.46	3.02 ± 0.33	2.36 ± 0.54	Nd
limonene	13.38	Nd	0.33 ± 0.05	0.33 ± 0.07	0.37 ± 0.03	2.15 ± 0.61	0.25 ± 0.23	0.23 ± 0.02
octanal	13.32	Nd	1.18 ± 0.26	1.14 ± 0.23	1.39 ± 0.27	1.09 ± 0.44	0.97 ± 0.26	1.41 ± 0.17
octane	8.43	Nd	1.30 ± 0.44a	1.01 ± 0.16a	0.82 ± 25a	1.04 ± 0.33a	2.47 ± 0.47b	4.16 ± 0.14c
trans,trans-2,4-decadienal	18.75	Nd	10.60 ± 3.82	10.73 ± 2.25	10.30 ± 1.91	10.91 ± 3.4	2.84 ± 0.04	1.89 ± 0.11
trans-2-decenal	17.81	Nd	7.05 ± 1.00	7.09 ± 2.39	7.84 ± 1.48	7.00 ± 3.60	5.26 ± 1.62	5.44 ± 0.48
dipropyldisulfide	15.08	Nd	14.88 ± 1.87	14.88 ± 1.87	12.01 ± 0.19	11.07 ± 2.54	Nd	Nd
trans-Citral	18.03	Nd	0.71 ± 0.29	0.71 ± 0.29	Nd	Nd	Nd	Nd
trans-propenylpropyldisulfide	15.27	Nd	2.42 ± 0.39	2.42 ± 0.39	2.62 ± 0.54	2.96 ± 1.07	Nd	Nd
Eucalyptol	13.61	Nd	Nd	Nd	Nd	16.37 ± 1.47	Nd	Nd
1-methyl-4-(1-methylethyl)-benzene	13.46	Nd	Nd	Nd	Nd	2.52 ± 0.44	Nd	Nd
2,4-heptadienal	13.91	Nd	Nd	Nd	1.66 ± 0.54	0.63 ± 0.08	0.48 ± 0.11	Nd
2-methyl-5-(1-methylethyl)-phenol	18.83	Nd	Nd	Nd	14.62 ± 4.14	Nd	Nd	Nd
4-terpineol	16.46	Nd	Nd	Nd	1.71 ± 0 41	2.5 2± 0.30	Nd	Nd
alpha-amorphene	20.44	Nd	Nd	Nd	Nd	0.63 ± 0.07	Nd	Nd
alpha-copaene	19.07	Nd	Nd	Nd	Nd	2.65 ± 0.37	Nd	Nd
alpha-humulne	20.40	Nd	Nd	Nd	Nd	0.48 ± 0.19	Nd	Nd
alpha-terpineol	16.76	Nd	Nd	Nd	2.46 ± 0.09	7.66 ± 1.32	Nd	Nd
beta-caryophyllene	19.92	Nd	Nd	Nd	3.69 ± 0.39	Nd	Nd	Nd
borneol L	16.61	Nd	Nd	Nd	21.40 ± 3.19	5.98 ± 1.07	Nd	Nd
camphene	11.95	Nd	Nd	Nd	Nd	0.59 ± 0.23	Nd	Nd
camphor	16.31	Nd	Nd	Nd	Nd	12.27 ± 2.62	Nd	Nd
delta-cadinene	20.98	Nd	Nd	Nd	0.91 ± 18	1.02 ± 0.42	Nd	Nd
linalool	15.01	Nd	Nd	Nd	4.43 ± 0.24	0.86 ± 0.34	Nd	Nd
myrcene	12.54	Nd	Nd	Nd	Nd	0.71 ± 0.16	Nd	Nd
thymol	18.60	Nd	Nd	Nd	1.47 ± 0.44	Nd	nd	Nd

## Supplementary Materials

The following are available online at https://www.mdpi.com/2076-3921/8/11/551/s1, Figure S1: Pareto chart of factors and interactions obtained from the BBD for the response, Table S1: Box–Behnken experimental design showing the levels of each independent variable (sample weight, extraction temperature, extraction time and NaCl concentration) for the HS-SPME extraction of target compounds in *sofrito* number 1.
