# Supplementary material for: Influence of Cooking and Ingredients on the Antioxidant Activity, Phenolic Content and Volatile Profile of Different Variants of the Mediterranean Typical Tomato Sofrito
Source: Antioxidants (Basel). 2019 Nov 14;8(11):551. doi: 10.3390/antiox8110551 (PMC6912581; doi:10.3390/antiox8110551)
Supplement: Supplementary file 1 [file antioxidants-08-00551-s001.pdf]

Supplementary Materials: Table S1. Box–Behnken experimental design showing the levels of each independent variable (sample weight, extraction temperature, extraction time and NaCl concentration) for the HS-SPME extraction of target compounds in *sofrito* number 1.

Run	Sample weight (g)	Temperature (ºC)	Time (min)	NaCl (M)
1	1.75	47.5	35	1
2	3	47.5	60	1
3	1.75	35	35	2
4	3	35	35	1
5	1.75	60	60	1
6	1.75	47.5	60	2
7	3	60	35	1
8	0.5	35	35	1
9	1.75	60	35	0
10	1.75	47.5	60	0
11	1.75	47.5	10	0
12	0.5	47.5	35	0
13	1.75	47.5	35	1
14	1.75	47.5	35	1
15	3	47.5	35	0
16	3	47.5	35	2
17	1.75	47.5	10	2
18	1.75	60	10	1
19	1.75	35	60	1
20	1.75	60	35	2
21	0.5	47.5	60	1
22	1.75	47.5	35	1
23	1.75	47.5	35	1
24	0.5	60	35	1
25	0.5	47.5	35	2
26	1.75	35	10	1
27	1.75	35	35	0
28	3	47.5	10	1
29	0.5	47.5	10	1

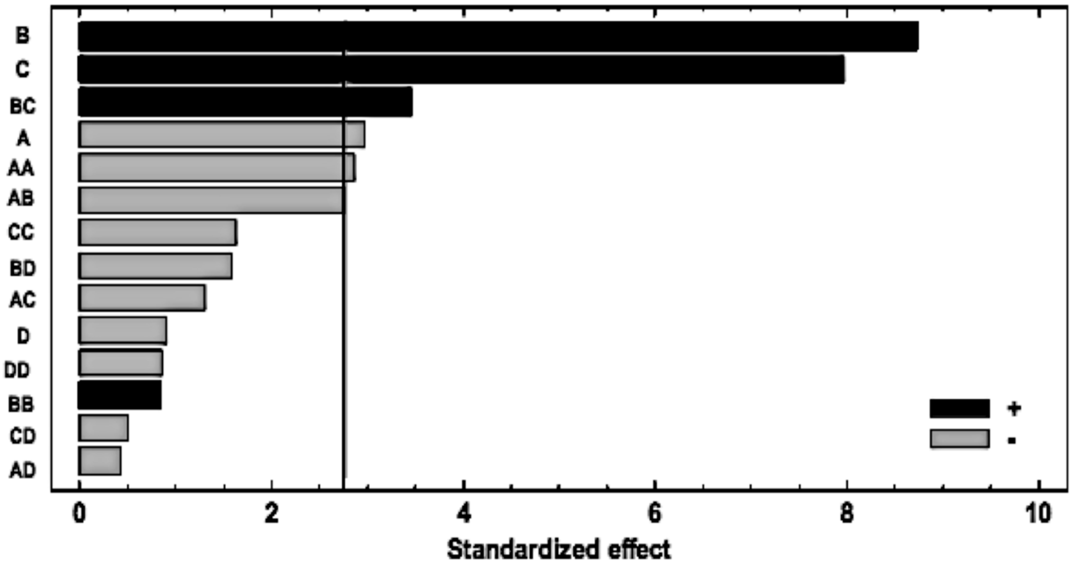

Supplementary Figure S1. Pareto chart of factors and interactions obtained from the BBD for the response.
